# Computational Tools and Techniques for Early Diagnosis and Screening of Geriatric Diseases

**DOI:** 10.1155/2018/7830584

**Published:** 2018-10-25

**Authors:** Hyuntae Park, Fimiharu Togo, Masashi Miyashita

**Affiliations:** ^1^Department of Health Care and Science, College of Health Science, Dong-A University, Busan, Republic of Korea; ^2^Educational Physiology Laboratory, Graduate School of Education, The University of Tokyo, Tokyo, Japan; ^3^Graduate School of Sport Sciences, Waseda University, Tokorozawa, Japan

Geriatric syndrome is defined as a set of multifactorial conditions associated with deficits in clinical, psychosocial, and environmental domains. Future care of the elderly should be based on the ability to recognize and quantitatively assess early predictors of geriatric syndrome at the primary healthcare level in order to prevent, or limit, the later development of geriatric syndrome. In cases where geriatric syndrome is established, quantitative techniques for monitoring the effectiveness of care and treatment of the disease are also necessary.

Usage of computational tools, new technical developments, and improvement in technologies involving wearable devices and sensors hold great promise for advancement of care systems for the elderly. Different academic disciplines have proposed a variety of meaningful advancements for both the initial screening and diagnosis and care of different age-related diseases and disorders. Future potential exists for early screening and diagnosis of geriatric syndrome based on evaluation of continuous time-series data acquired during daily life, using analytical methods rooted in current mathematical, statistical, and computer science concepts/techniques. Such development of computational tools would herald a new era for both modeling and optimizing treatments of such diseases as sarcopenia, cognitive decline, mood disorder, frailty, and dependency—each of which is considered as a geriatric syndrome. This special issue highlights a number of thoughtful viewpoints on the present application of such computational tools with the goal of evaluating the current status quo through publication of research articles and review papers on the topics above.

Topic of four papers is computational and statistical methods for disease early screening. J. Kim et al. provide a practical guide with SAS code of multilevel modeling for analyzing wrist activity data obtained by accelerometer and self-reported data from repeated measures using ecological momentary assessments in older adults. They show a possibility for early detection of increasing in depressive symptoms using wrist activity data during daily life. L. J. Mena et al. developed a mobile EEG monitoring system, which includes a machine learning classifier of normal and abnormal electrocardiogram signals for older adults. The system may be useful for detecting cardiac abnormalities during daily life. J. Beltrán et al. review on papers for eye movements during specific oculomotor tasks in older adults with Alzheimer's disease. They also introduce the progress in technology that can enable analyzing eye movements during daily life which may be useful for early detection of Alzheimer's disease. Y. Kim et al. propose an automatic drusen detection method using the median filter and Renyi's threshold algorithm in funds image. This method may help clinicians to improve the diagnostic performance in the detection of age-related macular degeneration.

Topic of one paper is wearable technology for disease early screening. M. H. Jang et al. introduce a newly developed surface electromyography device for monitoring muscle activity during daily life. This device may be useful in monitoring muscle activity during daily life and in preventing sarcopenia and frailty.

Topic of one paper is quantitative techniques for blood methylmercury concentration. B.-G. Kim et al. report statisticaly significant differences in values of the methylmercury in the same blood samples for older adutls between two analytic techniques (i.e., the dithizone extraction and gas chromatography-electron capture detector method and the cold vapor atomic fluorescence spectrophotometer method), indicating that these methods should be used carefully for evaluating methylmecury concentration in blood.

This special issue is selective. Among 12 submissions, 6 were selected. It is our hope that this impressive group of papers will help the analysis and treatment for older population in their efforts to advance community-based medical research.

